# Psychological Impact of a Prenatal Diagnosis of Congenital Heart Disease on Parents: Is It Time for Tailored Psychological Support?

**DOI:** 10.3390/jcdd11010031

**Published:** 2024-01-20

**Authors:** Giulia Mutti, Lamia Ait Ali, Marco Marotta, Silvia Nunno, Veronica Consigli, Stefania Baratta, Maria Letizia Orsi, Francesca Mastorci, Cecilia Vecoli, Alessandro Pingitore, Pierluigi Festa, Sabrina Costa, Ilenia Foffa

**Affiliations:** 1 Fondazione Toscana, G. Monasterio, Via Aurelia Sud, 54100 Massa, Italymarco.marotta@ftgm.it (M.M.); veronica.consigli@ftgm.it (V.C.); cecilia.vecoli@cnr.it (C.V.); gigifesta@ftgm.it (P.F.); sabrina.costa@ftgm.it (S.C.); ilenia.foffa@cnr.it (I.F.); 2 Istituto di Fisiologia Clinica CNR, Via Aurelia Sud, 54100 Massa, Italy; 3 Istituto di Fisiologia Clinica CNR, Via Moruzzi 1, 56124 Pisa, Italy; mastorcif@ifc.cnr.it (F.M.); pingi@ifc.cnr.it (A.P.)

**Keywords:** congenital heart defects, CHD, prenatal diagnosis, fear of childbirth, EMDR treatment, psychological impact of prenatal diagnosis on parents, post-traumatic stress, maternal psychological stress, psychological support, heart disease impact

## Abstract

The prenatal diagnosis of congenital heart disease (CHD) represents, for both parents, a particularly stressful and traumatic life event from a psychological point of view. The present review sought to summarize the findings of the most relevant literature on the psychological impact of prenatal diagnosis of CHD on parents, describing the most common mechanisms employed in order to face this unexpected finding. We also highlight the importance of counseling and the current gaps in the effects of psychological support on this population.

## 1. Introduction

Congenital heart defects (CHD) are the most common form of congenital malformation at birth [1]. Prenatal diagnosis improves CHD significantly with perinatal management, but also represents a challenge for parents who must learn to manage their child’s disease, which is often complex. In fact, early identification initially represents an emotional shock to the parents and a stressful event which can completely change their perception of pregnancy [2].

Therefore, the diagnosis of CHD firstly represents an unexpected event only later it can also represent an opportunity for parents to evaluate and use all available information to improve their decision-making with regard to their child.

Many studies evaluate the psychological impact of prenatal diagnosis of fetal malformation on parents’ psychological condition. Some of these focused on congenital heart disease [3,4,5,6,7,8,9,10,11]. They analyzed the reactions of the parents at diagnosis, during pregnancy, and after birth, showing low levels of psychological well-being. This evidence underlines the importance of psychological support during this process to facilitate an adjustment that helps them cope with the situation [3,4,12,13,14].

Another important aspect that should not be underestimated is how prenatal maternal stress can not only have negative effects in the short term, by altering fetal development, but can also compromise a child’s health and development in the long term [15]. This is probably due to epigenetic mechanisms and an altered release of 11ßHSD type 2 (HSD2), which usually is involved in the protection of the developing fetus from the adverse effects of excess maternal glucocorticoids [15,16,17].

In this regard, the Avon Longitudinal Study of Parents and Children showed that maternal stress during the last trimester of pregnancy is responsible for sleep disorders in children at 18 and 30 months of age, an altered emotional reactivity at the age of 4 years, attention deficit hyperactivity disorder (ADHD) symptoms, and, in adolescence, altered cognitive functions [18]. Thus, evidence has shown that parental mental health influences the emotional, behavioral, and developmental outcomes of healthy children [19]. 

Some authors highlight that with pathological conditions such as CHD, parental mental health is considered a stronger predictor of peripartum depression [20] and could be a trigger of emotional responses in children in addition to clinical factors [21].

A randomized controlled trial of psychological intervention in a CHD population showed that intervention promoted clinically significant gains for the child and family [22].

The current review attempts to summarize the findings of the most relevant literature on the psychological impact of prenatal diagnosis of CHD on parents, describing the most common mechanism employed in order to face this unexpected finding.

The importance of early and tailored psychological support in this population and the need to fill this gap are also highlighted. 

## 2. Emotive Reactions of Parents after Prenatal Diagnosis of Fetal Malformation

The prenatal diagnosis of fetal malformation is a stressful event affecting mood and emotions, resulting in an increase in anxiety level, depression symptoms, and stress perception for both parents, although research suggests that mothers and fathers could differ in their experience of stress. Carlsson [2] et al. presented an interesting model of the sequence of emotive reactions of parents after prenatal diagnosis of fetal malformation, articulating it in three phases:

- The first phase represents the moment of shock; the diagnosis is difficult to grasp for parents, and they shift from a normal and healthy pregnancy to a moment of confusion and unexpected personal tragedy [23]. The parents have no indications of an unhealthy fetus. Moreover, they can feel fetal movements, and this can make the diagnosis seem unreal. This phase involves the difficult decision about the opportunity to terminate the pregnancy or not, with all the future difficulties that this entails (existential thoughts, future suffering), highlighting the importance of sufficient information to reach a good decision [3,7].
- The second phase is described as the existential crisis characterized by emotions such as guilt, anger, pain, and unfairness. During this phase, it can become difficult to deal with everyday life (work, taking care of other children) and this can create a sense of inadequacy.
- The third phase is described as a remodeling phase in the lead up to the birth. The collected data show that in the more advanced stage of pregnancy and closer to the birth, the maternal bond with the child increases. A remodeling of one’s life takes place according to the needs of the child. This model explores the different stages gone through by the parental couple, from a psychological/emotional point of view., and could help to create interventions aimed at improving the well-being and support for parents at this delicate phase of life

An interesting and alarming aspect of the proposed model is the presence of worries that accompany the parents constantly, starting from concern about the health condition of the fetus (in the prenatal phase), to what will be the moment of birth (in the postnatal phase), with all that this entails. Furthermore, there is the prenatal medical follow-up, which parents will necessarily have to face with a different spirit from that of the initial phase.

Concerns about the condition of the fetus turn into worries about the baby, i.e., about what the baby’s health will be like after birth and accepting separation from one’s baby at an early stage because he or she will need intensive care or undergo surgery within days of birth, which puts a strain on both parents. One aspect that should not be underestimated is the worry of having to cope with the premature death of the infant. In parallel to these thoughts, is the aspect of organizing life at home and everything involved in managing a newborn who has undergone surgery and needs more attention and care.

Although much of the research conducted has focused on the role of stress and the emotional component experienced by parents following fetal malformation diagnosis, little attention has been paid to management actions implemented by parents through the utilization of coping strategies, usually based on their personal resources (emotional, cognitive, economic). For example, higher levels of acceptance and management were associated with low levels of anxiety, depression, and traumatic stress [5]. Additionally, fewer studies have focused on parental coping following postnatal diagnosis compared to prenatal CHD diagnosis, thus not considering the temporal aspect that is linked to the prenatal stress suffered by the mother and transmitted to the fetus via the placenta [24]. Starting from the moment of the inauspicious diagnosis, a completely different chapter in the lives of the parents is opened by inevitably focusing attention on aspects that, until recently, were totally unknown and unexplored.

## 3. Psychological Impact of Prenatal Diagnosis of CHD in Parents

We provide a detailed list of the most relevant evidence on the impact of prenatal diagnosis of CHD in parents (Table 1).

In summary, Rychik et al. [5] reported higher scores of stress, depression, and states of anxiety in pregnant women with a fetus with CHD in comparison with women with a healthy fetus. Moreover, a lower partner satisfaction was associated with higher depression and higher anxiety. In line with this, Bratt et al. [8] found that pregnant women with prenatal diagnosis of CHD scored higher for symptoms of depression compared to control and this persisted post-partum, although was not significantly different in comparison with mothers with post-natal diagnosis of CHD. Moreover, they presented a lower sense of coherence during pregnancy and lower life satisfaction after their childbirth. Bevilacqua et al. did not find significant differences in emotional distress, depression, and quality of life in parents with a diagnosis of prenatal or postnatal CHD, evaluated postnatally [10], which is in line with the study by Solberg et al. [23]. However, mothers who had received a prenatal diagnosis were more depressed, whereas those with a postnatal diagnosis were more stressed; fathers showed the same tendency. A possible explanation is in the fact that those who received prenatal diagnosis, after an initial moment of anger, disbelief, and stuntedness, had more time to organize themselves and cope with the problems and the stress load, while those who received the diagnosis later faced a totally unexpected situation. 

Furthermore, Davey et al. [4] reported that mothers of infants with a prenatal diagnosis of CHD presented significantly lower rates of life stress than mothers of infants with postnatal diagnosis of CHD, despite the higher severity of heart defects. Salvador et al. [3] found that both parents of fetuses with CHD had clinically significant scores of psychological distress after prenatal diagnosis; both parents went through stages of shock and denial in response to the diagnosis of disease, and this was mildly higher in the mothers. Carlsson et al. [11] interviewed 12 expectant fathers of fetuses with major CHD, including those who decided to terminate the pregnancy. They expressed strong emotion and stressed the importance of an informed joint decision regarding whether to continue or terminate the pregnancy. Solberg et al. underline that mothers of children with severe CHD also had higher depression scores than mothers of infants with mild or moderate CHD, and these findings persisted at 36 months postpartum. Mothers of children with severe CHD still report significantly higher symptoms of depression and anxiety compared with controls and with mothers of children with mild or moderate CHD [7,25]. This is in line with the studies that have found that anxiety and other maternal stress levels are closely correlated with diagnostic uncertainty and the severity of any fetal anomaly [9].

## 4. Impact of Maternal Stress on Outcome

- It is well established that prenatal maternal stress has adverse effects on pregnancy outcomes (i.e., preeclampsia, spontaneous abortion, and preterm delivery) [26]. Moreover, a growing literature suggests that prenatal maternal depression, anxiety, or stress is associated with cortical thinning, altered amygdala and hippocampus growth, and altered brain microstructure and functional connectivity in the offspring [6], shown in Table 1. According to the perspective of “Fetal programming of adult disease”, an early plasticity in cells and tissues is mediated by epigenetic adaptation that occurs during fetal life in response to environmental stimuli, and there even seems to be a transgenerational epigenetic inheritance showing effects induced by prenatal environment [27]. The association between intrauterine stress exposure and development of brain structure and function in the case of CHD is not completely clear, but previous studies suggest that impaired fetal hippocampal and cerebellar development are connected with stress, anxiety, and depression levels in pregnant women with CHD diagnosis [6]. One possible explanation could be related to the protective role of 11ßHSD type 2 (HSD2) in healthy conditions, while stress conditions cause an altered development of the HPA axis of the fetus. Another important aspect is the timing of prenatal stress. In fact, the type of effect on the fetus depends on the moment in pregnancy when the mother experiences the stress. In the first trimester, maternal stress is predominantly associated with coronary artery disease, dyslipidemia, and hypertension; in the second trimester stress may lead to pulmonary disorders; whereas stress in the last trimester mainly leads to behavioral effects [28].
- Maternal stress that can arise following the diagnosis of fetal anomaly and continue throughout the pregnancy, up to delivery and postpartum, and can also interfere with breastfeeding [29].

## 5. Psychological Support for Parents during Pregnancy and after Birth

**Figure 1:**
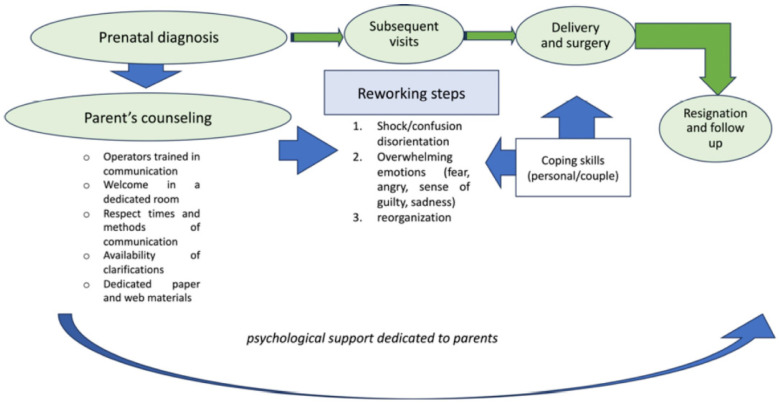
Psychological impact of prenatal diagnosis of fetal anomaly in parents.

Chronic diseases, such as CHD, are often managed throughout the lifespan, with the need to modify lifestyle and expectations due to the limitations related to the disease. In view of this, parents, especially in childhood and when their children become adolescents, must be supported in the management of the disease in those dimensions that are not strictly related to clinical outcomes but are fundamental to the child’s development, such as social and relational dimensions. In order to be able to do this, parents must not only know the clinical impact of the disease on their child, but also the possible impact on a psychological level and on learning [30] (Figure 1). 

To our knowledge, there is no relevant study that evaluates the impact of a tailored and early psychological support for parents after the prenatal diagnosis of CHD. However, many studies highlight the importance of early psychological support for parents, and in particular for mothers, to reduce maternal prenatal stress and optimize disease management [14]. Zhang et al. [31] suggested that confusion and lack of understanding about the diagnosis of CHD can bring stress to the family, and for this reason early psychological support combined with education about the disease is fundamental to help families. Although there is no evidence on this subject, one possibility would be to promote Behavioral Cardiology for patients’ disease management [32]. In this case, for parents, one would have to integrate health and emotional counseling, stress management techniques such as relaxation techniques, coping skills, mindfulness protocols, goal setting and motivational techniques, self-efficacy, self-monitoring, implementation actions, and social support like group education/management, as well as support programs.

As a matter of fact, a psychological intervention during hospitalization and, in particular, after/during admission to the intensive care unit for parents of CHD, contributes to reducing the level of anxiety, improving confidence, and parents coping, and this can also improve parents’ satisfaction with the clinic clinical care [33]. In a review, Woolf-King et al. [25] confirmed the need to transform screenings to assess mental health into a routine, standardized activity that is naturally incorporated into the path of pediatric care. This would allow for more careful monitoring of the evolution of any psychological problems, their severity, and the identification of the stages of greatest risk for parents and children [25]. 

Moreover, one study underlines the need for and benefit that can derive from a psychological intervention for the parents of children with CHD referred to the psychological service. It improves both coping skills and parenting styles, but also has a positive impact on the children themselves [34].

As reported by Tesson et al. [35] “Family-centred care remains fundamental for pediatric care and specific policies and best-practice recommendations are needed to support parent-child relationships following diagnosis of critical and chronic childhood medical illness”.

The clinical psychology service located across the Sydney Children’s Hospital networks since 2010dedicated to CHD at has demonstrated over the years the importance of integrated psychological care. By intervening on behalf of both children and young people with heart disease and also taking care of their families, they demonstrate the importance and validity of integrated psychological assistance with a positive effect on mental well-being. Furthermore, creating targeted training for healthcare personnel has a great impact on provision of increasingly adequate and accessible care, reducing both the actual costs for the healthcare system and the psychological difficulties that patients may experience [36].

## 6. Cruciality of Counseling

The American Heart Association (AHA) underlines the relevance of counseling and psychosocial support after a prenatal diagnosis of CHD [37].

The Association for European Pediatric and Congenital Cardiology (AEPC), among others, has outlined specific recommendations for the practice of fetal cardiology [38].

Carlsson T. et al. [39] sustain that early, realistic, and honest information is a fundamental factor in effective counseling, with special attention paid to non-native speakers. In this context, illustrations can be used to communicate and efficiently explain diagnosis and its implications, constituting the perfect vehicle for making medical information understandable and complementing oral information. Finally, it is important to create internet content with the right information to help parents in avoiding overexposure to excessive and sometimes erroneous information. It has been found that often the information obtained regarding the possibility of interrupting pregnancy is insufficient and unclear, both from health professionals and on the internet [40]. This may be because interruption represents a social stigma, and this could influence the content of the information. Moreover, when medical interruption of pregnancy would be the most recommended medical option in incurable fetal cardiac pathologies, it is fundamental to respect the patient’s will [41], considering the psychological effects of this decision in future life.

With this perspective, it is crucial to augment specialists’ communication skills with specific training sessions [42] for improving the communication of bad news to this vulnerable population [43]. Parents are particularly aware of ultrasound examination because it is a tool for identifying fetus anomalies, so they need to be prepared for the ultrasound examination, and the specialist performing the examination must be prepared for the parents’ anxiety load. Physicians are not always trained to maintain empathy and to provide emotional support during prenatal counseling. Therefore, this means that they need adequate training to maintain the right focus on empathic communication and, at the same time, they also need to work on their own well-being [44]. This underlines the essentiality of a professional support in the normal routine of examination [45].

Kovacevic et al. [46] synthesized, the different scientific societies recommendations and summarized the results of studies evaluating counseling, and they proposed an optimized multidisciplinary setting of counseling based on the current knowledge.

## 7. Current Gaps and Future Directions

Prenatal diagnosis allows for early identification of CHD in the fetus, significantly improving perinatal management; however, it represents, for both parents, a particularly stressful and traumatic life event.

Therefore, the optimization of care, community, and peer support can help the parents after a prenatal diagnosis of CHD and this could contribute to the future development of vulnerable children [47]. 

Bonnet argues that beyond this evidence much work remains to be done in concert, interacting and sharing across disciplines to improve knowledge in this area [47]. For example, there is growing evidence of the effects of a diagnosis of malformation of the fetus in the mother, but there are still few studies that include or are focused on fathers, and even fewer on siblings. There are, however, interesting studies regarding the experience of fathers in the case of premature birth of the child, in which the need to consider not only the emotional difficulties experienced by mothers but also those that emerge in fathers, that may be more hidden and silent but equally present, is emphasized [48]. Regarding the experience of siblings, i.e., in particular, siblings of children with CHD, Parker et al. [49] summarizes how much the birth of a child with CHD impacts on different aspects of the life of “healthy” siblings and how, for this reason, it is important to pursue studies and research in this area as well.

Moreover, a focused, early psychological intervention could improve fetal outcome.

Interestingly, a study on the effect of prenatal CHD diagnosis carried out on 140 women (48 diagnosed with CHD and 98 with a healthy fetus) by Wu et al. showed that maternal stress and anxiety were prevalent among women carrying a fetus with CHD, and this is associated with altered cerebellar and hippocampal development [6]. This reinforces the importance of universal screening for maternal psychological distress; a tailored psychological intervention could be helpful. For instance, Eye Movement Desensitization and Reprocessing (EMDR) has been used as an approach to prevent and reduce symptoms of post-traumatic stress disorder (PTSD) in women who have had a traumatic birth experience [50], or as an approach during pregnancy to augment personal resources and reduce stress and anticipatory anxiety, in relation to specific pain or potentially traumatic events, although not specifically related to a fetal anomaly or CHD diagnosis [51].

In any case, there is a need for more focused studies to determine which type of methodological approach is most effective to assist parents after a prenatal diagnosis of CHD. 

In summary, the diagnosis of fetal malformation as CHD is a stressful and traumatic life event for the parents. The complexity of the information related to fetal malformation and future management leads to parents’ confusion, with a cascade of emotive reactions. To face this unexpected tragedy, they need professional support in the form of adequate counseling by a trained physician and perhaps also a tailored psychological intervention. In this context, the “human factor” acquires capital importance [52]. They need a professional referral team that guides them in the necessary multi-disciplinary management, which is available to respond to their questions in understandable terms, and, ideally, which may share moments of discouragement and help in making difficult choices while respecting their wishes. This is the gap that ideally needs to be filled. There is a great need for humanity and personalization care in a highly technological word also aimed to the economic “sustainability” of the system.

## 8. Conclusions

There is growing evidence of the psychological impact of the prenatal diagnosis of CHD on the mother and on the father. Improving education, counseling, and stress management could help parents to cope with this traumatic event. According to a multilayer and multisectoral approach, one possibility could be to consider parents’ experience up to that moment; their degree of perceived well-being; their quality of life in its various dimensions, social, emotional, and psychological; to understand, given the enormous individual variability linked to the perception of traumatic events, what type of approach to implement; and to assess what type of intrinsic ability the parent has to manage such an event. However, there are still no clear and evidence-based guidelines on how to psychologically support both parents during pregnancy and the perinatal period. 

## Figures and Tables

**Table 1:** Impact of prenatal diagnosis on CHD in parents.

Ref.	Method	Population Study	Main Findings
Solberg et al. 2012 [7]	- Eight-item version of the Hopkins Symptom - CheckList-25 Assessed at regular intervals from pregnancy up to 36 months postpartum	- Mothers of infants with mild, moderate, or severe CHD - Control group	Severe CHD in infants had prolonged negative effects on the mothers’ mental health. Prevailing, heightened symptoms of depression and anxiety were identified compared to pregnancy cohort controls at 6, 18, and finally at 36 months postpartum.
Bratt et al. 2019 [8]	- The Swedish version of the hospital anxiety and depression scale - The Swedish version of sense of coherence scale - Life satisfaction questionnaire - Dyadic Adjustment Scale	Three groups: (1) Pregnant women and their partners with a diagnosis of CHD in the fetus (2) Parents of children with postnatally diagnosed CHD (3) Pregnant women with a normal screening ultrasound	- The prenatal diagnostic group scored higher for symptoms of depression compared to control during pregnancy and had a lower sense of coherence. - Postnatally, the prenatal and postnatal diagnostic groups had higher levels of anxiety compared to controls. - Life satisfaction was lower in the prenatal diagnostic group compared to that in the postnatal group and in controls.
Kaasen et al. 2017 [9]	Self-report questionnaires - General health Questionnaire-28 - Impact of Event Scale-22 (IES) - Edinburgh Postnatal Depression Scale	- Fetal anomalies: CHD - Control group	The fetal anomaly group scored significantly higher (especially on depression-related questions) compared to the control group.
Wu et al. 2020 [6]	- Perceived Stress Scale - Spielberger State-Trait Anxiety Inventory - Edinburgh Postnatal Depression Scale - Fetal MRI	- Pregnant with CHD fetuses - Control group	Depression scores were higher among 17 women carrying fetuses with single-ventricle CHD vs. 31 women carrying fetuses with two-ventricle CHD. Psychological distress in women with fetal congenital heart disease appears to be prevalent. Maternal stress was associated with impaired fetal cerebellar and hippocampal development during the second half of gestation.
Salvador et al. 2022 [3]	- Brief Symptoms Inventory-18 (BSI-18) - Dyadic Adjustment Scale (DAS) - Family Adaptability and Cohesion Evaluation Scale, version III (FACES-III)	- Pregnant with fetus with CHD and their partners - Control group	35.1% of fathers and 47.4% mothers had clinically significant scores of psychological distress.
Bevilacqua et al. 2013 [10]	The Italian version of postnatal questionnaire - General Health Questionnaire-30 (GHQ-30) - Beck Depression Index II - Health Survey-36 (SF-36)	- Parents of infants with prenatal diagnosis of CHD - Parents of infants with postnatal diagnosis of CHD	No difference was found between prenatal and postnatal groups in any field tested but, according to percentage, mothers receiving prenatal diagnosis were more depressed, whereas those receiving postnatal diagnosis were more stressed. Fathers showed same tendency.
Rychik et al. 2013 [5]	- Impact of Events Scale-Revised - Beck Depression Index II - State-Trait Anxiety Index - COPE Inventory - Dyadic Adjustment Scale	- Pregnant mothers of fetuses with CHD	Clinically important traumatic distress was seen in 39%, depression in 22%, and anxiety in 31%. Lower partner satisfaction was associated with higher depression (*p* < 0.01) and higher anxiety (*p* < 0.01).
Carlsson et al. 2018 [11]	Semi-structured interview	Twelve expectant fathers of fetuses with major CHD	The respondents experienced emotional distress in connection to the diagnosis and emphasized the importance of an informed joint decision.
Davey et al. 2023 [4]	- Symptom Inventory (BSI) - Impact of Events Scale–Revised (IES-R) assessing posttraumatic stress - Brief COPE - Center for Epidemiologic Studies Depression Scale (CES-D) - Life Stress Questionnaire	- Prenatal diagnosis of CHD - Postnatal diagnosis of CHD	A total of 68.6% had significant life stress, while 25.7% had clinical concerns or met criteria for Post-Traumatic Stress Disorder. Mothers of infants with a prenatal diagnosis of CHD reported significantly lower rates of life stress despite higher severity of heart defects.

## Data Availability

Not applicable.
